# Diet Influences the Oral Microbiota of Infants during the First Six Months of Life

**DOI:** 10.3390/nu12113400

**Published:** 2020-11-05

**Authors:** Patrícia M. Oba, Hannah D. Holscher, Rose Ann Mathai, Juhee Kim, Kelly S. Swanson

**Affiliations:** 1 Department of Animal Sciences, University of Illinois at Urbana-Champaign, Urbana, IL 61801, USA; obapm@illinois.edu; 2 Department of Food Science and Human Nutrition, University of Illinois at Urbana-Champaign, Urbana, IL 61801, USA; hholsche@illinois.edu; 3 Division of Nutritional Sciences, University of Illinois at Urbana-Champaign, Urbana, IL 61801, USA; 4 Department of Nutrition, Dominican University, River Forest, IL 60305, USA; rmathai@dom.edu; 5 Department of Nutrition, East Carolina State University, Greenville, NC 27834, USA; kim.juhee@gmail.com

**Keywords:** oral bacteria and fungi, infant health, feeding method

## Abstract

*Background*: Oral microorganisms contribute to oral health and disease, but few have studied how infant feeding methods affect their establishment. *Methods*: Infant (*n* = 12) feeding records and tongue and cheek swabs were collected within 48 h of birth, and after 2, 4, and 6 mo. DNA was extracted from samples, bacterial and fungal amplicons were generated and sequenced using Illumina MiSeq, and sequences were analyzed using Quantitative Insights Into Microbial Ecology (QIIME) and Statistical Analysis System (SAS) to evaluate differences over time and among breast-fed, formula-fed, mixed-fed, and solid food-fed infants. *Results*: Considering all time points, breast milk- and mixed-fed infants had lower oral species richness than solid food-fed infants (*p* = 0.006). Regardless of feeding mode, species richness was lower at birth than at other time points (*p* = 0.006). Principal coordinates analysis (PCoA) of unique fraction metric (UniFrac) distances indicated that bacterial communities were impacted by feeding method (*p* < 0.005). Considering all time points, breast-fed infants had higher *Streptococcus*, while formula-fed infants had higher *Actinomyces* and *Prevotella*. Regardless of feeding mode, *Propionibacterium*, *Porphyromonas*, *Prevotella*, *Gemella*, *Granulicatella*, *Veillonella*, *Fusobacterium*, *Leptotrichia*, *Neisseria,* and *Haemophilus* increased with age, while *Cloacibacterium* and *Dechloromonas* decreased with age. Oral fungi were detected in infants but were not impacted by diet. *Conclusions*: These findings demonstrate that the establishment of oral bacteria depends on dietary composition and age. More research is necessary to determine whether this affects risk of oral caries and other health outcomes later in life.

## 1. Introduction

Breast milk is the first food that most newborn humans come in contact with. Human milk not only contains nutrients, but is rich in microbes and oligosaccharides that contribute to the early colonization of the gastrointestinal tract [1]. The breast milk microbiome can vary throughout lactation [2], but *Streptococcus*, *Staphylococcus*, *Serratia, Pseudomonas*, *Corynebacteria*, *Ralstonia*, *Propionibacterium*, *Sphingomonas*, and *Bradyrhizobiaceae* are the predominant genera present [3]. Infant saliva and breast milk of mother-child pairs both have a high abundance of *Streptococcus* spp. [4], with breast milk serving as an important source of microorganisms for the infant. However, around 5 mo of age, an infant’s oral microbiota differs from maternal microbiota and consists mostly of *Streptococcus*, *Haemophilus*, *Neisseria*, and *Veillonella* [5].

Clinical evidence suggests that the microbial colonization may begin in utero [6,7,8,9] and that delivery method influences the original seeding of microorganisms [10,11]. Therefore, the delivery mode affects the gastrointestinal tract microbiota of the infant [12,13,14], with vaginally delivered infants, at 3 mo of age, having an oral microbiota with higher taxonomic diversity than cesarean-born children [12]. Feeding method also affects the oral microbiota. There appear to be differences between breast-fed and formula-fed infants, with possible reasons for this being that breast-fed infants have higher lactobacilli populations that are capable of inhibiting the growth of *Streptococcus* [15]. Milk microbiota at 3–4 mo postpartum is dominated by Proteobacteria and Firmicutes, and milk microbiota composition and diversity are associated with maternal factors (body mass index, parity, and mode of delivery), breast feeding practices, and other milk components [16].

Information about fungal acquisition and maturation of the oral mycobiome is limited. The *Candida* genus is the most prevalent yeast in the oral cavity, with others in high abundance including *Cladosporium*, *Aureobasidium*, *Saccharomycetales*, *Fusarium*, *Cryptococcus*, and *Aspergillus* [17,18,19]. *Candida* spp. can be acquired by the infant through vertical transmission from the mother’s vagina during birth [14,20]. Furthermore, it was reported that the increase of oral *Candida* load is associated with a low bacterial diversity, a dominance of Bacilli (streptococci and lactobacilli) and disappearance of Fusobacteria and Bacteroidia [21].

Oral bacteria and fungi contribute to oral health and establishment of the distal gastrointestinal tract microbiota [22,23,24,25]. However, there is a dearth of knowledge on the longitudinal changes that occur during the first few mo of life and how feeding method affects oral bacteria and fungi in infants. Therefore, we aimed to characterize the relationships between feeding method and oral bacterial and fungal communities in vaginally delivered infants during the first 6 mo of life. We hypothesized that the alpha diversity of the microbiota would increase over time, most notably with the addition of formula and/or solid foods. We hypothesized that (1) the infant oral bacterial and fungal microbiome would have a greater diversity with time, (2) the oral bacterial and fungal microbiome of each infant would have a greater diversity with the change from formula feeding to solid foods, and (3) similar to previous studies, *Streptococcus* would be the predominant oral bacterial genera in infants at all time points.

## 2. Materials and Methods

### 2.1. Study Design

This was a longitudinal, observational trial. Study procedures were administered in accordance with the Declaration of Helsinki and were approved by the University of Illinois at Urbana-Champaign Institutional Review Board (IRB #11621; approved on 14 September 2011) and the Carle Foundation Hospital Institutional Review Board (IRB #11020; approved on 7 December 2011) prior to participant recruitment. Healthy pregnant women who intended to breast feed their infants were recruited during their third semester of pregnancy (wk 26–40) in collaboration with the Carle Hospital Midwives. Healthy pregnant women were defined as women with uncomplicated pregnancy, such as those without preeclampsia, gestational diabetes, anemia, and no history of smoking. Infants were included in the study if they were vaginally delivered at 37 wk of gestation or later. Study exclusion criteria included: cesarean section delivery, smoking during pregnancy, or any serious medical condition (e.g., preeclampsia, eclampsia).

### 2.2. Study Participants and Sample Collection

Samples from tongue and cheek were collected using a Catch-All Sample Collection Swab buccal swab (Epicentre Biotechnologies, Madison, WI), from a cohort of infants (*n* = 12) at four time points: at birth (within 48 h after delivery) and at 2 mo, 4 mo, and 6 mo of age. Two tongue samples were collected by brushing the center of the dorsum for 5 s and then placed into a sterile tube. Using separate swabs, two cheek samples were collected using a similar method. All samples were placed on ice, immediately transported to the laboratory, and then stored at −80 °C until analyses. Feeding practices were separated as exclusive human milk, formula, mixed feeding (human milk + formula), and introduction to solid foods (mixed of solid foods + human milk/formula or both).

### 2.3. DNA Extraction and Sequencing

Sample DNA was extracted from all samples individually using the MoBio PowerSoil PowerLyzer kits (Mo Bio Laboratories, Inc., Carlsbad, CA, USA) according to manufacturer guidelines with minor modifications conducted in consultation with the manufacturer. Briefly, to increase DNA yield, columns were washed with 125 μL of solution C2 and 125 μL of solution C3 at the same time, rather than two steps, and a low elution spin filter (MoBio) was utilized to elute the DNA. After extraction, DNA quality was assessed using an E-Gel system (Invitrogen, Carlsbad, CA, USA) followed by quantification using a Qubit 2.0 Fluorometer (Life Technologies, Grand Island, NY, USA). Amplification of the variable region 3–5 (V3–V5) of the bacterial 16S rRNA (ribosomal ribonucleic acid) gene [26] and fungal internal transcribed spacer (ITS) 1–4 region [27] as conducted on a Fluidigm Access Array (Fluidigm Corporation, South San Francisco, CA, USA); CS1 forward tag and CS2 reverse tag were added according to the Fluidigm protocol. Quality of the amplicons was assessed using a Fragment Analyzer (Advanced Analytics, Ames, IA, USA) to confirm amplicon regions and sizes. A DNA pool was generated by combining equimolar amounts of the amplicons from each sample. The pooled samples were then size selected on a 2% agarose E-gel (Life technologies) and extracted using a Qiagen gel purification kit (Qiagen, Valencia, CA, USA). Cleaned, size-selected, pooled products were run on an Agilent Bioanalyzer (Advanced Analytics, Ames, IA, USA) to confirm appropriate profile and average size. Illumina sequencing was performed on a MiSeq using v3 reagents (Illumina Inc., San Diego, CA, USA) at the W. M. Keck Center for Biotechnology at the University of Illinois.

### 2.4. Data Analyses

Forward reads were trimmed using the FASTX-Toolkit (version 0.0.13), and Quantitative Insights Into Microbial Ecology (QIIME) 1.8 [28] was used to process the resulting sequence data. Briefly, high-quality (quality value ≥ 20) sequence data derived from the sequencing process were demultiplexed. Sequences then were clustered into operational taxonomic units (OTU) using UCLUST [29] through a closed-reference OTU picking strategy against the Greengenes 13_8 reference database [30] with a 97% similarity threshold. Singletons (OTU that were observed fewer than two times) and OTU that had less than 0.01% of the total observation were discarded. Taxonomic identity to each OTU was then assigned using UCLUST. An even sampling depth (sequences per sample) of 30,882 sequences per sample was used for assessing alpha diversity [observed species; phylogenetic diversity (PD_whole_tree); Chao1] and beta diversity measures. Beta diversity was calculated using weighted and unweighted unique fraction metric (UniFrac) [31] distance measures. Fungal sequences were picked using the pick_open_reference_otus.py command and the ITS_12_11_otus reference taxonomy provided by the UNITE database (https://unite.ut.ee).

All samples were collected, stored, and extracted separately. The samples were combined at the data analysis step because initial analysis showed that there were no statistical differences between the two points of collection. All data were analyzed using Statistical Analysis System (SAS, version 9.4, SAS Institute, Cary, NC, USA) using the Mixed Models procedure with dietary treatment and period being the fixed effect and infant being the random effect. Due to lack of samples for all feeding groups at all times, a time*diet interaction was not tested. When analyzing the main effect of diet, infants were separated into two groups: exclusive breast feeding until 4 mo (*n* = 6) and non-exclusive breast feeding until 4 mo (*n* = 6). Data normality was checked using the univariate procedure and Shapiro-Wilk statistic, with log transformation being used when normal distribution was lacking. If the data did not reach normality after a logarithmic transformation, the data were analyzed using the npar1way procedure and Wilcoxon statistic. Data were reported as means with *p* < 0.05 being considered significant. As this was a preliminary trial, there were no corrections for multiple testing [32].

## 3. Results

A total of 10,899,630 16S rRNA-based amplicon sequences were obtained, with an average of 113,538 reads (range = 31,336–175,809) per sample, rarefied at 30,882. On Table S1, there is the dietary information of infants at each time point and on Table S2, additional information of infants/mothers and delivery complications. Due to the lack of statistical differences between sample types, cheek and tongue sequence data were combined.

### 3.1. Effect of Time on Infant Oral Microbiota

Time had a significant influence on the oral bacterial phyla (Table 1), with Firmicutes being the phyla of highest prevalence in the infant oral cavity, accounting for 78.8 ± 26.3% of all sequences. Firmicutes were higher (*p* = 0.0002) at 2 mo than other time points, followed by 4 mo, 6 mo, and birth that was the lowest. Actinobacteria were higher (*p* = 0.005) at birth compared to 4 mo and 6 mo. Bacteroidetes was higher (*p* < 0.0001) at 6 mo compared to birth and 2 mo, with 4 mo having an intermediate value. Fusobacteria were higher (*p* < 0.0001) at 6 mo than birth, 2 mo, and 4 mo, with 2 mo and 4 mo being greater than birth when these taxa were virtually absent. Proteobacteria were very high at birth, being higher (*p* < 0.0001) than 2 mo, 4 mo, and 6 mo. Proteobacteria were also higher (*p* < 0.0001) at 6 mo compared to 2 and 4 mo.

*Streptococcus* spp. were by far the genera of highest prevalence in the infant oral cavity, accounting for 64.1 ± 27.2% of all sequences (Table 2). In general, older infants had a higher abundance of *Actinomyces*, *Porphyromonas*, and *Prevotella* of the Prevotellaceae and Paraprevotellaceae families, unclassified Weeksellaceae, *Gemella*, *Granulicatella*, unclassified Streptococcaceae, unclassified Lachnospiraceae, *Veillonella*, *Fusobacterium*, *Leptotrichia*, *Neisseria,* and *Haemophilus*. In contrast, *Cloacibacterium*, unclassified Gemellaceae, unclassified Comamonadaceae, and *Dechloromonas* were more abundant at birth and, in general, decreased with age. *Streptococcus* was the only genus that had a lower concentration at birth and 6 mo and higher at 2 and 4 mo, with the opposite pattern observed with *Propionibacterium* and *Actinobacillus*, which had a higher percentage at birth and 6 mo and lower at 2 and 4 mo (Table 2).

**Figure 1:**
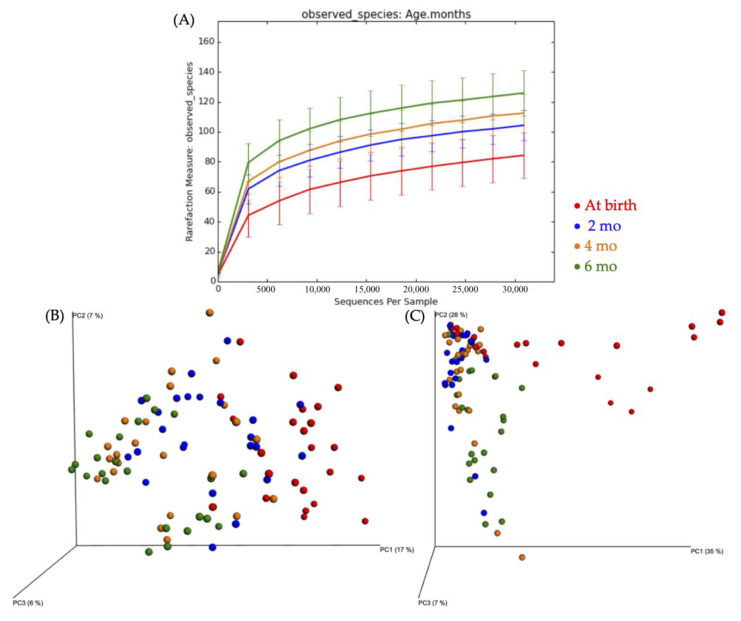
(**A**) Rarefaction curves showing bacterial species richness based on infant age. Mean number of operational taxonomic units (OTU) per subject for infant age. (**B**) Unweighted principal coordinates analysis (PCoA) plot of identified bacterial OTUs, shows plot for infant age. (**C**) Weighted PCoA plot of identified bacterial OTUs, shows plot for infant age.

Species richness differed between birth and 2 mo (*p* = 0.006), birth and 4 mo (*p* = 0.006), birth and 6 mo (*p* = 0.006), 2 mo and 6 mo (*p* = 0.006), and 4 mo and 6 mo (*p* = 0.018; Figure 1A), but did not differ between the 2 mo and 4 mo (*p* = 0.144). Unweighted and weighted principal coordinates analysis (PCoA) for infant age tended to cluster birth and 6 mo separately (Figure 1B,C).

Oral fungal communities were impacted by infant age (Table 3). Five infants had detectable levels of fungi within 24–48 h of birth, which changed to four, nine, and 10 at 2, 4, and 6 mo of age, respectively. Ascomycota counts were higher (*p* = 0.0475) at 2 mo of age than 4 and 6 mo of age, with infants at birth having intermediate values. Ascomycota was present in infants even within the first 24–48 h after birth (detected in 41.67% of infants in at least one site of collection), including 33% of infants by the age of 2 mo, 67% of infants by the age of 4 mo, and 83% of infants by the age of 6 mo. Basidiomycota was detected in 33% of infants at birth, 17% of infants at 2 mo, 33% of infants at 4 mo, and 58% of infants at 6 mo. *Candida* spp. were detected at a low frequency at birth (detected in 17% of infants in at least one site of collection), in 25% of infants at 2 and 4 mo, and 75% of infants by the age of 6 mo.

### 3.2. Effect of Diet on Infant Oral Microbiota

Length of breast feeding and exclusive breast feeding until for 4 mo did not have a significant influence on the bacterial phyla populations at any age (Table 4). When comparing the effect of receiving breast milk as an exclusive source of food within the first 24–48 h after birth, our results revealed that infants exclusively breast-fed had lower *Haemophilus* concentrations than those receiving a mix of formula and milk (Table S3). Overall, exclusively breast-fed infants had higher *Neisseria* (*p* = 0.01) and *Actinobacillus* (*p* = 0.0001) at birth, *Streptococcus* (*p* = 0.02) by the age of 2 mo, and unclassified Weeksellaceae (*p* = 0.0001) abundance by the age of 6 mo when compared to infants receiving mixed feedings. However, infants receiving only breast milk had lower *Veillonella* (*p* = 0.01) abundance by the age of 2 mo than those receiving mixed feedings (Table 5). Infants who received solid foods had higher abundance of *Gemella* (*p* = 0.0003), unclassified Streptococcaceae (*p* < 0.0001), *Veillonella* (*p* < 0.0001), *Fusobacterium* (*p* = 0.006), and *Neisseria* (*p* = 0.0004). *Streptococcus* (*p* = 0.02), *Actinobacillus* (*p* = 0.003), and unclassified Gemellaceae (*p* = 0.02) were higher for breast-fed infants and lower for solid food-fed, and *Actinomyces* (*p* = 0.0002), *Prevotella* (*p* < 0.0001), and unclassified Lachnospiraceae (*p* < 0.0001) were higher for formula-fed infants (Table 6).

**Figure 2:**
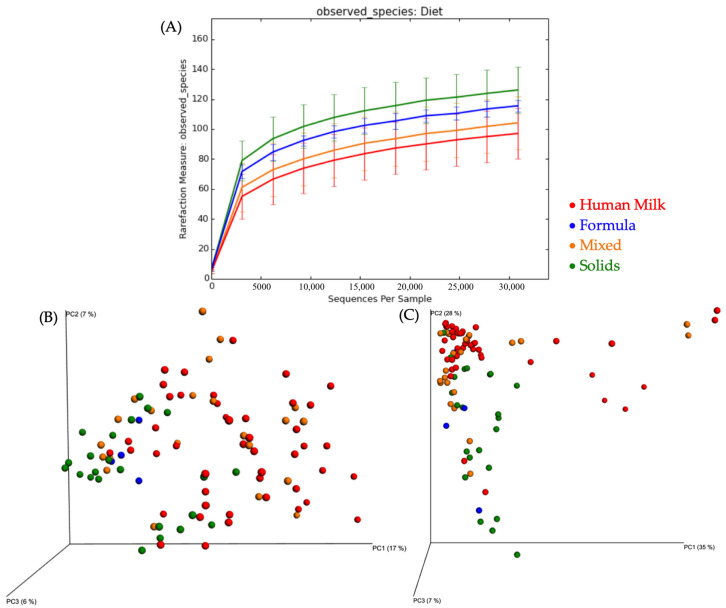
(**A**) Rarefaction curves showing bacterial species richness based on feeding method. Mean number of bacterial operational taxonomic units (OTU) per subject for feeding method. (**B**) Unweighted PCoA plot of identified bacterial OTUs, shows plot for feeding method. (**C**) Weighted PCoA plot of identified bacterial OTUs, shows plot for feeding method.

Considering all time points, the species richness differed between the solid food- vs. human milk-fed groups (*p* = 0.006) and solid food- vs. mixed-fed groups (*p* = 0.006; Figure 2A). Species richness did not differ between the human milk- and mixed-fed groups, solid- and formula-fed groups, mixed- and formula-fed groups, or human milk- and formula-fed groups. Unweighted and weighted PCoA for feeding method tended to cluster the solid food feeding method separately, and with human milk being separate from mixed feeding method (Figure 2B; Figure 2C).

**Figure 3:**
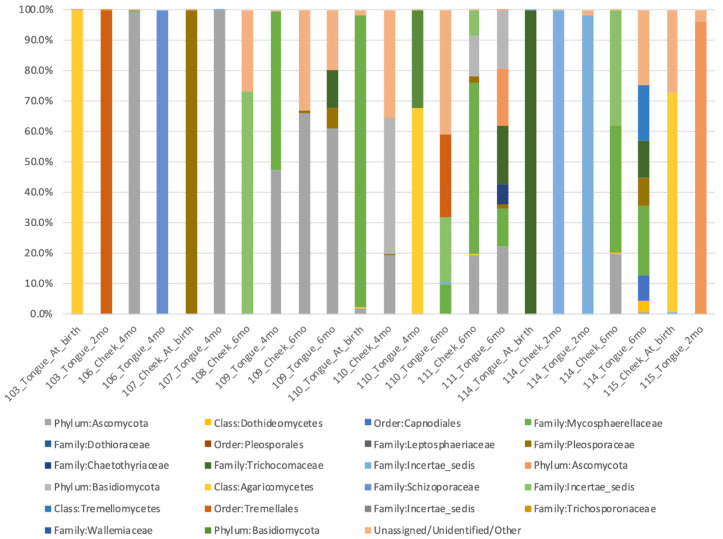
Fungal community (>5% classified) composition was diverse in infants.

Fungal communities of all samples are represented on Figure 3. Fungal communities were not impacted by diet (Table 7). Ascomycota and Basidiomycota were not detected in infants fed formula. Ascomycota was detected in 52% of infants fed human milk (in at least one point and site of collection), 36% of infants fed mixed diet, and 83% of infants fed solid foods. Basidiomycota was detected in 39% of infants fed human milk ((HM) in at least one point and site of collection), 9% of infants fed mixed diet, and 58% of infants fed solid foods. *Candida* spp. were detected at a low frequency in infants fed human milk (detected in 22% of infants in at least one point and site of collection), in 27% of infants receiving mixed diet, and in a higher frequency of infants fed solids foods (detected in 75% of infants).

## 4. Discussion

Studies using high-throughput sequencing methods to examine the oral microbiome of infants within 6 mo of age are rare [5,11]. To our knowledge, the current study is the only one using this technology to report relationships between feeding method and age on both bacterial and fungal communities in the infant oral cavity during this early stage of life.

As demonstrated in previous studies, the oral bacterial communities of infants were dominated by the phylum Firmicutes [5,33,34], being similar to results from children (3 to 6 years old) [35] and adults [36,37] in previous studies. Proteobacteria were the second most abundant phyla, being similar to previous studies in adults (>27%) [36] and infants (>8%) [5]. The main difference between the current study and a previous study with infants (mean age = 4.6 ± 1.2 mo old) [5], children [35], and adults [37] was the higher prevalence of Bacteroidetes. In those populations, these phyla were present at an average relative abundance of 16–37%. In the current study, it was only 7.16 ± 9.77% of total sequences and more similar to that of Cephas et al. [5]. Cephas et al. [5] study used the V4–V6 region of 16S rDNA genes, Ling et al. [35] used the V3 region of 16S rRNA genes, and Keijser et al. [37] used the V6 region of 16S rRNA genes, and all were subjected to 454-pyrosequencing. In the present study, the V3–V5 regions of 16S rRNA genes were used and subjected to Illumina sequencing, which may contribute to the reported differences.

At the genus level, our results are in agreement with previous studies of the oral microbiota of infants ≤6 mo of age. Similar to the previous studies, the predominant genera present in samples of infants was *Streptococcus* spp. [5,33,34], which were detected in all infants from the ages of 2 to 24 mo [38]. The present study also detected *Streptococcus* spp. as the predominant genera and all infants and ages tested had the presence of this genus. In agreement with the current study, infants of 4–5 mo of age had a predominance of *Streptococcus*, *Veillonella*, *Haemophilus*, and *Granulicatella* being observed at average levels >1% of sequences [5]. In contrast to the previous study in our laboratory, the infants of 4–5 mo of age did not have *Propionibacterium*, *Porphyromonas*, *Prevotella*, or *Leptotrichia* present at average levels >1% of sequences [5]. Moreover, *Neisseria, Rothia*, *Gemella*, *Leptotrichia*, and *Fusobacterium* were not present in the current study, but were predominant genera in 4-mo-old infants in the previous study. Furthermore, *Veillonella*, *Fusobacterium*, *Streptococcus*, and *Actinomyces*, which are considered to be early colonizers of the oral cavity [39,40,41], were all detected in the oral cavity of infants tested in the present study in all periods (at birth, 2 mo, 4 mo, and 6 mo of age). However, in birth, *Actinomyces*, *Fusobacterium*, and *Veillonella* have a low number of species in comparison with all the other periods.

Previous research has shown that the most abundant genera in human milk are *Streptococcus* and *Staphylococcus* [3,42]. *Lactobacillus* is also known to be a common member of the milk microbiota. In the present study, the most abundant genera in the oral cavity of infants fed human milk were *Streptococcus*, *Actinobacillus*, *Neisseria*, *Veillonella,* and *Staphylococcus*. Moreover, *Lactobacillus* was isolated from 100% of breast-fed infants (for at least one site of collection) at birth, 86% at 2 mo, 83% at 4 mo, and in the only infant consuming human milk at 6 mo, however, at a low relative abundance. Breast milk most likely contributes to the population of *Streptococcus*, *Staphylococcus*, and *Lactobacillus* in the oral cavity of infants. Similar to previous studies, *Prevotella* spp. (phylum Bacteroidetes) were more abundant in the oral cavity of formula-fed infants when compared to breast-fed infants [33]. Additionally, it was reported that *Lactobacillus* spp. were not detected in formula-fed infants at the age of 3 mo [15]. Conversely, in the present study, *Lactobacillus* spp. were detectable in infants exclusively fed formula (*n* = 2) and in the majority of infants fed a mixture of formula and breast milk (4 of 5). Only two infants receiving a mixture of formula and breast milk lacked *Lactobacillus* spp. as part of their oral microbiota in one point of sampling (considering all points of samplings), but these genera were only present after 2 mo. when fed a mixture of formula and breast milk. One infant lacked oral *Lactobacillus* spp. when fed a mixture of formula and breast milk but had it when receiving exclusive breast milk or solid food. In a previous study, *Bifidobacterium* spp. were detected in the oral cavity of only one breast-fed (1 of 26) and two formula-fed (2 of 12) infants [33]. Similar in the current study, *Bifidobacterium* spp. were not measured in any of the mouths of breast-fed or formula-fed infants.

Children and young adults with dental caries have been reported to have higher concentrations of *Veillonella*, *Lactobacillus*, *Bifidobacterium*, *Propionibacterium*, low-pH non-*S. mutans* Streptococci, *Actinomyces*, and *Atopobium*. These bacteria are thought to impact the development and progression of dental caries [43]. Similarly, *Actinomyces*, *Granulicatella*, *Veillonella*, *Bifidobacteriaceae*, and *Scardovia* have been identified as contributors to early childhood caries [44,45,46]. In the present study, infants fed human milk exclusively for 4 mo had lower *Veillonella* concentrations by the age of 2 mo than those receiving mixed feedings. This may indicate that a longer period of exclusive breastfeeding would potentially benefit oral health in early life, as previous studies have reported that breastfeeding in infancy may protect against dental caries [47].

Another factor that can affect the oral microbiota of infants is birth delivery mode. Within seconds after delivery, *Lactobacillus*, *Prevotella*, *Atopobium*, and *Sneathia* spp. have been reported to be the most predominant oral bacteria in vaginally delivered infants [11]. In the present study, all infants were vaginally delivered. In the first samples, which were collected within 48 h after birth (Table S3), the predominant genera were *Streptococcus*, *Actinobacillus*, *Haemophilus*, *Neisseria*, *Cloacibacterium*, *Propionibacterium*, *Staphylococcus*, and *Dechloromonas*. Gram-positive cocci, including *Streptococcus* and *Staphylococcus*, have been reported to be some of the most frequent early colonizers of the oral cavity [48,49]. Our results suggest that even in a short period (within 48 h after delivery) the oral microbiota of infants changes drastically from *Lactobacillus*, *Prevotella*, *Atopobium*, and *Sneathia* spp. to those measured in our study, likely due to environmental exposures during the first few hours (e.g., provision of colostrum or formula, introduction of pacifiers, hospital conditions, contact with hospital staff and family, etc.). Another study reported that vaginally delivered infants at the age of 3 mo. had a higher proportion of *Capnocytophaga* (16%), *Prevotella* (40%), *Leptotrichia* (16%), and *Haemophilus* (28%) than caesarian section-delivered infants of the same age [12]. In the present study, 100% of children had detectable levels of oral *Haemophilus*, *Leptotrichia*, and *Prevotella* at 2 mo of age. Only three (25%) samples from the tongue and five (41.67%) samples from the cheek, however, were positive for *Capnocytophaga*. These data suggest that environmental exposures early in life are more influential than delivery mode.

Fungi may also play a role in oral health. *Candida* spp. have been reported to be the most predominant yeast in the oral cavity, having a high frequency (75%) in oral rinse samples collected from healthy adults (21–60 yr old), followed by *Cladosporium* (65%), *Aureobasidium* (50%), *Saccharomycetales* (50%), *Aspergillus* (35%), *Fusarium* (30%), and *Cryptococcus* (20%) [19]. All of those fungal genera, with the exception of *Cryptococcus* (phylum Basidiomycota), are part of the phylum Ascomycota. In another study comparing oral microbiota of healthy adults and adults with periodontal disease, it was reported that *Candida* and *Aspergillus* were the most frequently observed genera (isolated from 100% of participants), followed by *Penicillium* (97%), *Schizophyllum* (93%), *Rhodotorula* (90%), and *Gibberella* (83%). *Schizophyllum* and *Rhodotorula* are members of the phylum Basidiomycota, while the others are members of Ascomycota. Peters et al. [50] reported that differences in overall oral mycobiome diversity or composition were not observed between participants with periodontal disease and participants with good oral health, but that Ascomycota had a higher frequency of sequence reads (86.5%) in all oral wash samples. Human milk may contribute to the presence of fungi in the oral cavity. By comparing the human milk mycobiota extracted at two different points in time (7–15 days (transient milk) and 45–90 days (mature milk)), a recent study reported that the most abundant genera in transient milk were *Aspergillus* (44.2%), *Saccharomyces* (35.8%), and *Malessezia* (12.3%), and in mature milk they were *Aspergillus* (57.9%), *Penicillium* (18.6%), and *Malessezia* (10.5%) [51]. All of those fungal genera, with the exception of *Malessezia* (phylum Basidiomycota), are part of the phylum Ascomycota. To our knowledge, the current study is the first to evaluate the mycobiome diversity of infants from birth to 6 mo of life. Ascomycota and Basidiomycota were also measured in infants, even within the first 24–48 h after birth. In general, the frequency of yeast in the oral cavity increased with age. Additionally, *Candida* spp. were measured in a low frequency at birth, with a higher frequency of these yeasts as age increased. Furthermore, infants fed human milk or a mixture of human milk and formula had a lower frequency of *Candida* spp. than when infants were fed solid foods. These results may indicate that Ascomycota, Basidiomycota, and *Candida* spp. are regular members of the oral mycobiome, with effects coming from the type of diet consumed. More studies are needed to elucidate the role of yeast on oral health and the effects of food on the mycobiome.

This study evaluated bacteria and fungi in a longitudinal trial but did have a few limitations. Because a small number of infants were studied, and due to the experimental design and samples collected, this study cannot determine causality. Rather, it was only possible to identify associations among microbial taxa, infant age, and feeding strategy. In addition, no vaginal swabs, breast milk, and food offered (solid or formula) were collected. For this reason, it was not possible to correlate the contribution of the external microbial populations on the oral microbiota of infants. Finally, due to a lack of samples at each time point, testing diet × time interactions with sufficient power was not possible.

## 5. Conclusions

In conclusion, the current study has provided novel information about the oral microbiota of healthy human infants from between birth and 6 mo of age and how microbiota changes relate to feeding method. Differences were detected in the taxonomic profiles of different feeding methods, with infants receiving solid foods having higher concentrations of *Gemella*, *Veillonella*, *Fusobacterium*, *Neisseria*, and *Actinobacillus*. Considering all time points, breast-fed infants had a higher concentration of *Streptococcus*, and formula-fed infants had a higher concentration of *Actinomyces* and *Prevotella*. We also noted alterations in the oral microbiota over time, with increased abundance of *Propionibacterium*, *Porphyromonas*, *Prevotella*, *Gemella*, *Granulicatella*, *Veillonella*, *Fusobacterium*, *Leptotrichia*, *Neisseria,* and *Haemophilus* with age, and a decrease of *Cloacibacterium* and *Dechloromonas* with age. In addition, the older infants had a higher frequency of oral yeasts, namely, *Candida* spp. In addition, breast-fed and mixed-fed infants had a lower frequency of *Candida* spp. than those fed solids foods. Our data suggest that environmental exposures early in life are more influential than delivery mode.

## Figures and Tables

**Table 1:** Oral bacterial phyla over time in human infants (% of sequences).

Phyla	Period (*n* = 12)				Statistics	
	Birth	2 mo	4 mo	6 mo	SEM	Period *p*-Values
Actinobacteria	2.1 ^a^	1.7 ^ab^	1.4 ^b^	1.3 ^b^	0.85	0.005
Bacteroidetes	3.1 ^c^	3.7 ^c^	7.3 ^b^	14.5 ^a^	2.12	<0.0001
Firmicutes	59.2 ^z^	92.5 ^w^	87.6 ^x^	76.1 ^y^	5.24	0.0002
Fusobacteria	0.0 ^c^	0.5 ^b^	1.6 ^b^	2.5 ^a^	0.56	<0.0001
Proteobacteria	35.7 ^a^	1.5 ^c^	2.1 ^c^	5.6 ^b^	4.27	<0.0001

^a–c^ Means with different superscripts within a row differ by Tukey’s test (*p* < 0.05). ^w–z^ Means with different superscripts within a row differ by Wilcoxon’s test (*p* < 0.05).

**Table 2:** Oral bacterial genera of infants over time (% of sequences).

Phyla	Family	Genus	Period (*n* = 12)				Statistics	
			Birth	2 mo	4 mo	6 mo	SEM	Period *p*-Values
Actinobacteria	Actinomycetaceae	*Actinomyces*	0.0 ^c^	1.4 ^b^	1.3 ^a^	1.2 ^a^	0.41	<0.0001
	Propionibacteriaceae	*Propionibacterium*	2.1 ^w^	0.0 ^z^	0.0 ^y^	0.0 ^x^	0.66	0.006
Bacteroidetes	Porphyromonadaceae	*Porphyromonas*	0.0 ^c^	0.4 ^b^	1.611 ^b^	2.6 ^a^	0.49	<0.0001
	Prevotellaceae	*Prevotella*	0.0 ^c^	2.6 ^b^	3.050 ^a^	4.2 ^a^	1.45	<0.0001
	Paraprevotellaceae	*Prevotella*	0.0 ^c^	0.5 ^bc^	2.030 ^b^	5.6 ^a^	1.40	<0.0001
	Weeksellaceae	Unclassified	0.0 ^b^	0.3 ^a^	0.625 ^a^	1.6 ^a^	0.34	<0.0001
		*Cloacibacterium*	3.0 ^a^	0.0 ^b^	0.0 ^b^	0.0 ^b^	0.63	<0.0001
Firmicutes	Staphylococcaceae	*Staphylococcus*	1.8	0.7	0.5	0.6	0.34	0.460
	Gemellaceae	Unclassified	9.4 ^a^	4.0 ^a^	4.1 ^a^	4.2 ^b^	2.04	0.049
		*Gemella*	0.1 ^z^	0.1 ^y^	0.3 ^x^	0.7 ^w^	0.16	<0.0001
	Carnobacteriaceae	*Granulicatella*	0.0 ^d^	0.3 ^c^	1.6 ^b^	4.0 ^a^	0.42	<0.0001
	Lactobacillaceae	*Lactobacillus*	0.0	1.3	0.0	0.0	0.48	0.594
	Streptococcaceae	Unclassified	0.0 ^c^	0.0 ^c^	0.2 ^b^	0.4 ^a^	0.04	<0.0001
		*Streptococcus*	47.2 ^b^	78.9 ^a^	74.2 ^a^	56.1 ^b^	5.36	<0.0001
	Lachnospiraceae	Unclassified	0.3 ^z^	0.9 ^y^	0.6 ^x^	0.3 ^w^	0.41	0.021
	Veillonellaceae	*Veillonella*	0.0 ^b^	6.3 ^a^	5.7 ^a^	9.7 ^a^	1.40	<0.0001
Fusobacteria	Fusobacteriaceae	*Fusobacterium*	0.0 ^c^	0.3 ^bc^	0.4 ^ab^	0.9 ^a^	0.26	<0.0001
	Leptotrichiaceae	*Leptotrichia*	0.0 ^c^	0.1 ^c^	1.2 ^b^	1.6 ^a^	0.52	<0.0001
Proteobacteria	Comamonadaceae	Unclassified	8.5 ^a^	0.0 ^b^	0.0 ^b^	0.0 ^b^	1.79	<0.0001
	Neisseriaceae	*Neisseria*	3.3 ^b^	0.7 ^b^	0.3 ^b^	3.4 ^a^	0.99	<0.0001
	Rhodocyclaceae	*Dechloromonas*	1.6 ^a^	0.0 ^b^	0.0 ^b^	0.0 ^b^	0.33	<0.0001
	Pasteurellaceae	*Actinobacillus*	11.4 ^w^	0.0 ^y^	0.0 ^z^	0.0 ^x^	3.04	0.002
		*Haemophilus*	8.0 ^z^	0.5 ^y^	1.5 ^x^	1.9 ^w^	2.32	0.017

^a–d^ Means with different superscripts within a row differ by Tukey’s test (*p* < 0.05). ^w–z^ Means with different superscripts within a row differ by Wilcoxon’s test (*p* < 0.05).

**Table 3:** Infant oral fungi categorized over time (% of sequences).

Phylum	Period				Statistics	
	Birth	2 mo	4 mo	6 mo	SEM	Period *p*-Values
Ascomycota	42 ^ab^	83 ^a^	22 ^b^	26 ^b^	14.5	0.047
Basidiomycota	28	19.68	21	16	12.0	0.755
Unidentified	5	1	28	26	11.1	0.194

^a,b^ Means with different superscripts within a row differ by Tukey’s test (*p* < 0.05).

**Table 4:** Oral bacterial phyla of infants by age and duration of breast feeding (% of sequences).

Phyla	Exclusive Breast Feeding for at Least 4 mo (*n* = 6)				Breast-Fed for Less than 4 mo (*n* = 6)				Statistics	
	Period								SEM	Diet × Period *p*-Values
	Birth	2 mo	4 mo	6 mo	Birth	2 mo	4 mo	6 mo		
Actinobacteria	0.1	0.6	1.3	1.5	4.1	2.9	1.6	1.0	1.17	0.148
Bacteroidetes	3.4	1.1	1.9	11.3	2.8	6.4	12.8	17.7	2.80	0.194
Firmicutes	54.8	95.1	93.6	78.5	63.5	89.8	81.6	73.7	7.55	0.342
Fusobacteria	0.0	0.7	0.1	2.3	0.0	0.3	3.0	2.7	0.78	0.135
Proteobacteria	41.7	2.5	3.1	6.4	29.7	0.6	1.0	4.9	6.13	0.641

**Table 5:** Oral bacterial genera of infants by age and duration of breast feeding (% of sequences).

Phyla	Family	Genus	Breast-Fed at Least 4 mo (*n* = 6)				Breast-Fed for Less than 4 mo (*n* = 6)				Statistics	
			Birth	2 mo	4 mo	6 mo	Birth	2 mo	4 mo	6 mo	SEM	Diet × Period *p*-Values
Actinobacteria	Actinomycetaceae	*Actinomyces*	0.0	0.5	1.2	1.4	0.0	2.2	1.4	1.0	0.58	0.051
	Propionibacteriaceae	*Propionibacterium*	0.1 ^ab^	0.0 ^b^	0.0 ^b^	0.0 ^b^	4.0 ^a^	0.0 ^b^	0.0 ^b^	0.0 ^b^	0.90	0.003
Bacteroidetes	Porphyromonadaceae	*Porphyromonas*	0.0	0.6	0.4	3.2	0.0	0.1	2.8	2.1	0.69	0.129
	Prevotellaceae	*Prevotella*	0.0	0.1	0.2	0.6	0.0	5.1	5.9	7.9	1.82	0.051
	Paraprevotellaceae	*Prevotella*	0.0	0.0	0.1	4.5	0.0	0.9	3.9	6.8	2.01	0.436
	Weeksellaceae	Unclassified	0.0 ^d^	0.3 ^abc^	1.1 ^ab^	3.0 ^a^	0.1 ^cd^	0.2 ^c^	0.2 ^c^	0.2 ^b^	0.41	0.0001
		*Cloacibacterium*	3.2 ^ab^	0.0 ^bc^	0.0 ^bc^	0.1 ^bc^	2.7 ^a^	0.0 ^b^	0.0 ^c^	0.0 ^c^	0.92	0.009
Firmicutes	Staphylococcaceae	*Staphylococcus*	0.8	1.0	0.6	0.5	2.8	0.4	0.5	0.6	0.47	0.829
	Gemellaceae	Unclassified	14.0	3.6	4.6	3.7	4.7	4.4	3.5	4.6	2.87	0.348
		*Gemella*	0.0	0.0	0.5	1.2	0.2	0.1	0.1	0.3	0.22	0.082
	Carnobacteriaceae	*Granulicatella*	0.0	0.1	0.5	4.3	0.0	0.4	2.6	3.8	0.58	0.083
	Lactobacillaceae	*Lactobacillus*	0.0	0.5	0.0	0.0	0.0	2.0	0.0	0.0	0.68	0.936
	Streptococcaceae	Unclassified	0.0	0.0	0.1	0.4	0.0	0.1	0.3	0.3	0.06	0.370
		*Streptococcus*	39.8 ^c^	87.5 ^a^	84.7 ^ab^	60.9 ^bc^	54.5 ^bc^	70.3 ^bc^	63.7 ^bc^	51.3 ^c^	7.35	0.021
	Lachnospiraceae	Unclassified	0.0	0.0	0.0	0.2	0.7	1.8	1.2	0.5	0.56	0.156
	Veillonellaceae	*Veillonella*	0.0 ^d^	2.3 ^bc^	2.5 ^b^	7.2 ^ab^	0.0 ^cd^	10.3 ^a^	8.8 ^ab^	12.1 ^a^	1.70	0.010
Fusobacteria	Fusobacteriaceae	*Fusobacterium*	0.0	0.7	0.1	1.2	0.0	0.0	0.7	0.6	0.37	0.194
	Leptotrichiaceae	*Leptotrichia*	0.0 ^d^	0.0 ^d^	0.0 ^cd^	1.1 ^bc^	0.0 ^d^	0.2 ^cd^	2.3 ^bc^	2.2 ^ab^	0.72	0.025
Proteobacteria	Comamonadaceae	Unclassified	8.7 ^a^	0.0 ^b^	0.1 ^b^	0.1 ^b^	8.3 ^a^	0.0 ^b^	0.0 ^b^	0.0 ^b^	2.61	0.001
	Neisseriaceae	*Neisseria*	6.5 ^ab^	1.3 ^abc^	0.3 ^c^	4.4 ^a^	0.0 ^c^	0.2 ^b^	0.4 ^c^	2.5 ^a^	1.29	0.011
	Rhodocyclaceae	*Dechloromonas*	1.3 ^ab^	0.0 ^bc^	0.0 ^bc^	0.0 ^c^	1.8 ^a^	0.0 ^bc^	0.0 ^bc^	0.0 ^c^	0.49	0.010
	Pasteurellaceae	*Actinobacillus*	22.7 ^a^	0.0 ^bc^	0.0 ^b^	0.0 ^b^	0.0 ^b^	0.0 ^b^	0.0 ^b^	0.1 ^b^	4.03	0.0001
		*Haemophilus*	0.0 ^b^	0.7 ^ab^	2.5 ^ab^	1.9 ^a^	15.9 ^ab^	0.3 ^ab^	0.5 ^ab^	2.0 ^ab^	3.14	0.041

^a–d^ Means with different superscripts within a row differ by Tukey’s test (*p* < 0.05).

**Table 6:** Oral bacterial genera of infants by feeding method (% of sequences).

Phyla	Family	Genus	Diet				Statistics	
			Human Milk	Formula	Mixed	Solids	SEM	Diet *p*-Values
Actinobacteria	Actinomycetaceae	*Actinomyces*	0.8 ^z^	1.5 ^w^	0.8 ^y^	1.3 ^x^	0.44	0.0002
Bacteroidetes	Prevotellaceae	*Prevotella*	0.2 ^z^	14.8 ^w^	2.0 ^y^	5.3 ^x^	1.26	<0.0001
	Weeksellaceae	Unclassified	0.0	0.9	0.7	1.7	0.40	0.105
Firmicutes	Staphylococcaceae	*Staphylococcus*	1.1	0.2	1.0	0.5	0.35	0.609
	Gemellaceae	Unclassified	5.8 ^a^	2.9 ^ab^	6.6 ^ab^	4.0 ^b^	2.14	0.023
		*Gemella*	0.0 ^y^	0.3 ^z^	0.3 ^x^	0.7 ^w^	0.18	0.0003
	Streptococcaceae	Unclassified	0.0 ^b^	0.4 ^ab^	0.2 ^b^	0.4 ^a^	0.05	<0.0001
		*Streptococcus*	68.4 ^w^	67.3 ^y^	65.1 ^x^	54.4 ^z^	5.99	0.021
	Lachnospiraceae	Unclassified	0.2 ^z^	5.5 ^w^	0.5 ^y^	0.4 ^x^	0.32	<0.0001
	Veillonellaceae	*Veillonella*	2.5 ^z^	5.1 ^y^	6.9 ^x^	9.7 ^w^	1.40	<0.0001
Fusobacteria	Fusobacteriaceae	*Fusobacterium*	0.1 ^b^	0.5 ^ab^	0.5 ^ab^	0.8 ^a^	0.29	0.006
Proteobacteria	Neisseriaceae	*Neisseria*	2.3 ^x^	0.1 ^y^	0.1 ^z^	3.3 ^w^	0.95	0.0004
	Pasteurellaceae	*Actinobacillus*	5.4 ^x^	0.2 ^z^	0.5 ^y^	0.7 ^w^	3.11	0.003

Human Milk—exclusively fed with human milk fed, Formula—exclusively fed with formula, Mixed—fed with human milk + formula, Solids—fed with solid foods + human milk/formula or both. ^a,b^ Means with different superscripts within a row differ by Tukey’s test (*p* < 0.05). ^w–z^ Means with different superscripts within a row differ by Wilcoxon’s test (*p* < 0.05).

**Table 7:** Infant oral fungal populations by feeding method (% of sequences).

Phylum	Diet			Statistics	
	Human Milk	Mixed	Solids	SEM	Diet *p*-Values
Other	4.9	3	11	7.4	0.20
Ascomycota	28	66	34	12.0	0.19
Basidiomycota	28	0	20	9.5	0.47
Unidentified	18	17	23	10.0	0.29

Human milk—exclusively fed with human milk, Mixed—fed with human milk + formula, Solids—fed with solid foods + human milk/formula or both.

## Supplementary Materials

The following are available online at https://www.mdpi.com/2072-6643/12/11/3400/s1, Table S1: Dietary information of infants at each time point, Table S2: Additional information of infants/mothers, and delivery complications, Table S3. Influence of feeding method on bacterial genera at birth (24 – 48 h after delivery).
