# Supplementary material for: Diet Influences the Oral Microbiota of Infants during the First Six Months of Life
Source: Nutrients. 2020 Nov 5;12(11):3400. doi: 10.3390/nu12113400 (PMC7694519; doi:10.3390/nu12113400)
Supplement: Supplementary file 1 [file nutrients-12-03400-s001.pdf]

Supplementary Materials:

Table S1. Dietary information of infants at each time point

ID#	Birth	2 mo	4 mo	6 mo
101	human milk	human milk	human milk	human milk, formula, and solid
103	human milk	human milk	human milk	human milk
104	human milk and formula	human milk and formula	human milk and formula	human milk, formula, and solid
106	human milk and formula	formula	formula and solid	formula and solid
107	human milk	human milk	human milk and formula	human milk and solid
108	human milk	human milk	human milk	human milk and solid
109	human milk	human milk and formula	human milk and formula	human milk, formula, and solid
110	human milk	human milk	human milk	human milk and solid
111	human milk	human milk	human milk	human milk
112	human milk and formula	human milk and formula	formula	formula and solid
114	human milk	human milk and formula	human milk and formula	human milk, formula, and solid
115	human milk	human milk	human milk	human milk and solid

Table S2. Additional information of infants/mothers, and delivery complications

ID#	Term[1]	Delivery complications[2]	APGAR Score[3]	Infant weight at birth	Infant height at birth	Prescribed antibiotic/antifungal for mom	Prescribed antibiotic/antifungal for infant
101	Full term	GBS positive	1 minute - 9<br>5 minute - 9	3572 g	50.8 cm	Ampicillin - DOB	
103	Full term	GBS positive<br>Nuchal cord	1 minute - 8<br>5 minute - 9	3118 g	49.5 cm	Vancomyin -BOB	
104	Full term	Nuchal cord<br>Cyanotic	1 minute - 7<br>5 minute - 9	4819 g	58.4 cm		
106	Early term	Nuchal cord<br>Mild thrombocytopenia	1 minute - 4<br>5 minute - 7	3459 g	50.2 cm		
107	Early term	None	1 minute - 8<br>5 minute - 9	4451 g	52.1 cm		Amoxicillin - 3 days
108	Full term	Nuchal cord	1 minute - 8<br>5 minute - 9	3374 g	50.8 cm		
109	Full term	None	1 minute - 9<br>5 minute - 9	4054 g	54.6 cm		
110	Late term	None	1 minute - 8<br>5 minute - 9	4082 g	52.1 cm		
111	Late term	None	1 minute - 8<br>5 minute - 9	3884 g	54.6 cm		
112	Full term	GBS positive<br>Nuchal cord	1 minute - 6<br>5 minute - 9	3799 g	52.1 cm	Clindamycin - DOB	
114	Full term	GBS positive	1 minute - 8<br>5 minute - 9	3600 g	54 cm	Ampicillin - DOB	Nystatin - 3mo
115	Early term	Hemorrhage	1 minute - 8<br>5 minute - 9	3544 g	52.1 cm		

[1]Early term: Your infant is born between 37 wk, 0 d and 38 wk, 6 d; Full term: Your infant is born between 39 wk, 0 d and 40 wk, 6 d; Late term: Your infant is born between 41 wk, 0 d and 41 wk, 6 d.

[2]GBS = Group B streptococcal.

[3]Apgar score [1] includes color, heart rate, reflexes, muscle tone, and respiration. Scores of 7 to 10 are considered reassuring.

Table S3. Influence of feeding method on bacterial genera at birth (24 – 48 h after delivery)

Phyla	Family	Genus	Diet[1]		Statistics	
			Human milk	Mixed	SEM	P-values
Actinobacteria	Actinomycetaceae	*Actinomyces*	0.002	0.002	0.0004	0.8492
	Propionibacteriaceae	*Propionibacterium*	2.669	0.227	1.5023	0.3820
Bacteroidetes	Porphyromonadaceae	*Porphyromonas*	0.005	0.005	0.0012	0.9976
	Prevotellaceae	*Prevotella*	0.012	0.008	0.0015	0.1422
	Paraprevotellaceae	*Prevotella*	0.009	0.009	0.0012	0.8774
	Weeksellaceae	Unclassified	0.003	0.124	0.0367	0.0696
		Cloacibacterium	3.671	0.786	2.2667	0.5678
Firmicutes	Staphylococcaceae	*Staphylococcus*	1.633	2.280	0.9048	0.7752
	Gemellaceae	Unclassified	9.424	9.144	6.4588	0.5938
		Gemella	0.019	0.454	0.1381	0.2501
	Carnobacteriaceae	*Granulicatella*	0.008	0.029	0.0070	0.2453
	Lactobacillaceae	*Lactobacillus*	0.002	0.002	0.0005	0.5170
	Streptococcaceae	Unclassified	0.015	0.003	0.0100	0.8191
		Streptococcus	47.165	47.230	15.2445	0.9980
	Lachnospiraceae	Unclassified	0.458	0.003	0.4813	0.6892
	Veillonellaceae	*Veillonella*	0.027	0.053	0.0080	0.2301
Fusobacteria	Fusobacteriaceae	*Fusobacterium*	0.001	0.001	0.0004	0.8034
	Leptotrichiaceae	*Leptotrichia*	0.003	0.004	0.0005	0.2142
Proteobacteria	Comamonadaceae	Unclassified	10.335	2.986	6.6671	0.7782
	Neisseriaceae	*Neisseria*	4.339	0.052	2.1681	0.2571
	Rhodocyclaceae	*Dechloromonas*	1.790	0.892	1.1606	0.9396
	Pasteurellaceae	*Actinobacillus*	15.156	0.021	11.2180	0.3861
		Haemophilus	0.075[b]	31.587[a]	6.3867	0.0150

[1]Human milk = exclusively fed human milk, mixed = mixed human milk and formula fed.

References

1. Apgar, V. A. Proposal for a new method of evaluation of the newborn infant. *Anesth. Analg.* **2015**, *120*, 1056–1059, doi:10.1213/ANE.0b013e31829bdc5c.
